# Contraception Choice for Female Endurance Athletes: What’s Sport Got to Do With It? A Cross-Sectional Survey

**DOI:** 10.1007/s40279-024-02078-1

**Published:** 2024-09-01

**Authors:** Stephanie Ryall, Heidi Ohrling, Trent Stellingwerff, Stephanie Black, Kristen Reilly, Jane S. Thornton

**Affiliations:** 1 Return to Health and Performance Lab, Department of Family Medicine, Schulich School of Medicine & Dentistry, Western University, London, ON Canada; 2 Canadian Sport Institute Pacific, Victoria, BC Canada; 3 Department of Obstetrics & Gynecology, Western University, London, ON Canada; 4 Department of Family Medicine, Western University, London, ON Canada

## Abstract

**Background:**

While there are several prescribed contraceptive methods available, limited evidence exists to guide contraceptive decision-making in the context of endurance sport.

**Objectives:**

Study objectives were to characterize current and previous use, perceived impacts, and the decision process around contraceptives in endurance athletes.

**Methods:**

This was an online survey study with female endurance athletes recruited through social media and emails to university/club coaches and sport organizations. Quantitative and qualitative data were analyzed with descriptive statistics and conventional content analysis, respectively.

**Results:**

A total of 323 female endurance athletes participated. Among current contraception users (*n* = 182), 51% used hormonal intrauterine devices (hIUDs), 29% oral contraceptive pills (OCPs), and 13% nonhormonal IUDs (nhIUDs). hIUD users had the highest perceived positive training (39%) and competition (29%) impacts, citing reduced menstrual bleeding and symptoms as positive side effects. OCP and nhIUD users had higher rates of perceived negative training impacts (OCPs 10%, nhIUDs 30%). For OCP users, 31% reported perceived adverse body composition outcomes and 37% reported negative mood changes. Among nhIUD users, 74% experienced heavier, more irregular menstrual bleeding. Over half of participants were unsure about the impact of their current method on performance. For contraceptive selection, 95% felt that information from physicians was important, yet 32% felt performance was inadequately considered during counseling discussion. Athletes reported less frustration with their contraception choice when counseled in the context of sport.

**Conclusions:**

This exploratory study quantifies and qualifies the lived experiences of female endurance athletes with contraception. hIUDs were the most currently used and well-tolerated contraceptives among participants. This research offers valuable insights for athletes seeking contraception and looking to optimize both performance and health, along with the healthcare professionals guiding them.

## Key Points

**Table:** 

Hormonal IUDs were the most commonly used and best-tolerated contraceptive method for female endurance athletes in this study. This is a key finding as there are no previous studies on the impact of hormonal IUDs on athletic performance.
Frustration was the most frequent emotion athletes experienced when selecting a contraceptive, mainly due to the trial-and-error process, lack of evidence, and feelings of dismissal from physicians regarding athletic concerns.
Athletes whose physicians provided counseling in the context of sport were more likely to feel confident and well informed in their decision.

## Introduction

Selecting the right method of contraception is challenging for many women owing to the considerable number of contraceptives available with different mechanisms of action, methods of use, pregnancy prevention efficacy rates, contraindications, and side-effect profiles. Types of prescribed contraceptives^1^ that require consultation with a healthcare professional include combined oral contraceptive pills (OCPs), progestin-only OCPs, hormonal intrauterine devices (IUDs), nonhormonal (copper) IUDs, injections, patches, rings, and implants along with permanent surgical options [1].^2^ Selection is further complicated as contraceptives are not used solely for pregnancy prevention but can also be treatments for medical conditions such as heavy menstrual bleeding, dysmenorrhea, endometriosis, polycystic ovarian syndrome, and severe acne vulgaris [2–5]. Finding a suitable option may involve the sequential trial of multiple types of contraceptives [6]. For female endurance athletes, the decision has an added layer of significance as they must consider the potential impact of contraceptives on their athletic training and performance.

Little is currently known about how different contraceptives impact endurance sport. Most of the available evidence on athletics and contraception centers around OCPs and is not specific to the endurance athlete population [7–14]. In one of the most comprehensive systematic reviews (*n* = 42) on this topic, Elliot-Sale et al. (2020) concluded that OCPs might minimally decrease athletic performance, but group effects were likely trivial [15]. Of note, 83% of the studies in the review provided moderate-, low-, or very-low-quality evidence, and most studies focused on untrained or recreationally active women and measured nonendurance exercise outcomes. Additionally, while there has been research conducted that explores athletes’ experiences with contraceptives [12–14], these studies did not report on experiences specific to the endurance athlete population. Of the few studies that focused on endurance athletes, there was no stratification of experiences between different forms of hormonal contraception [7, 16] and no studies to date on the impact of IUDs on endurance performance. Consequently, practical conclusions cannot be drawn about different contraceptives for the female endurance athlete population.

This topic is important because of the high rates of use; studies on elite female athletes across a diverse array of sport disciplines have reported the prevalence of lifetime hormonal contraceptive use to be 70% and current use to be 50–57% [7, 13, 17]. There are no studies that specifically report the prevalence of prescribed contraceptive use in competitive endurance athletes, nor any studies that include both hormonal and nonhormonal methods.

To address these gaps, a cross-sectional survey study was designed to explore female endurance athletes’ experiences with prescribed contraception. The three main objectives were to characterize: (1) which contraceptives female endurance athletes are currently using or have previously used; (2) the perceived impact of contraceptives on health and performance in training and competitions; and (3) the contraception selection process including patient–physician interactions, questions and concerns, and resources consulted. These subjective cross-sectional data are a first step in informing future contraception selection guidelines for female endurance athletes.

## Methods and Analysis

A cross-sectional exploratory survey was developed, piloted, and delivered anonymously online via Qualtrics software (Qualtrics, Seattle). Data were collected between August 2021 and March 2022. Ethics approval was obtained from Western University’s Research Ethics Board (no. 119351).

### Study Population and Recruitment

Female (≥ 18 years of age) endurance athletes were recruited, with “endurance” defined as any sport requiring continuous high-intensity, primarily aerobic, exertion for a period of approximately 4 min or longer [18]. Respondents must have considered using, be currently using, or have previously used prescribed contraception. They must have also considered how contraceptives could impact their athletic performance. Individuals who considered but did not use contraception were included in the study to better characterize objective 3 as their responses provided a more complete understanding of the factors, questions, and concerns athletes had in the contraception selection process, along with patient–physician experiences. There were no restrictions on athlete nationality or competition level in the eligibility criteria. Participation was limited to individuals aged 18 and older primarily to prevent concerns regarding minors providing consent for the survey. Additionally, this age limitation was applied as adults are more likely to have made deliberate decisions about their participation in sport and contraceptive choices.

Participants were recruited via a combination of targeted and snowball sampling [19]. Recruitment emails were sent to Usports (the National University sport governing body in Canada), National Collegiate Athletics Association (NCAA, the United States’ intercollegiate athletics governing body), and club endurance coaches, along with Canadian endurance sport organizations. The emails introduced the author team, briefly outlined the objectives of the study, and asked if they would consider distributing the study invitation and recruitment poster to their female athletes. Social media recruitment posts were also shared on Twitter, Instagram, and Facebook.

### Survey Design

The survey questions were initially conceptualized and written by the co-first authors, as guided by the study objectives. No previous surveys were used as a basis. The first draft was reviewed for accuracy, comprehensiveness, and flow by the collaboration team, which included an exercise physiologist (T.S.), an obstetrician gynecologist (S.B.), an experienced researcher (K.R.), and a sport medicine clinician scientist (J.S.T.). Feedback was received and incorporated. The survey was then piloted by two female endurance athletes at Western University to ensure the wording of questions and answers were easy to follow and there were no technical difficulties with the Qualtrics platform. Minor adjustments were made on the basis of their suggestions. The survey underwent a final round of revisions following submission to ethics.

The survey contained multiple choice, Likert scale, and open-ended text questions on four main topics: athlete demographics, contraceptive consideration process, contraceptive usage history, and physician counseling experiences. Within the demographics section, athlete level was categorized as either provincial, national, or international level. These terms were defined for participants as follows: ‘Provincial/state level’ if you competed for your club/team/school at a provincial/state championship and/or at competitions around the province/state; ‘National level’ if you competed for your club/team/school at a national championship and/or at competitions around the country; and ‘International level’ if you competed for your country and/or sponsor in international competitions.

Pilot testing indicated that the survey took approximately 15–30 min to complete. Prior to starting the survey, participants were required to read the Letter of Information, provide electronic consent, and answer three mandatory eligibility questions. If they did not meet the criteria, the survey automatically ended. Adaptive questioning was enabled with skip logic; therefore, the total number of questions presented to each participant varied. The minimum number of questions was 25 for those who considered but never used contraception. The survey contained 46 questions for those who used one contraceptive, and the length increased by 13 questions for each additional method used so participants could share the positive and negative side effects and perceived impact on athletic performance for each. The open-ended questions were followed by blank text boxes with no word limit. Participants were able to navigate forwards and backwards in the survey, and all questions beyond the eligibility criteria were optional.

No personal identifiers were collected, and all responses were anonymized.

### Statistical Analysis

Descriptive statistics were used to analyze quantitative data from multiple choice and Likert scale questions. This was performed using a combination of Microsoft Excel (Microsoft, Redmond WA) and pandas, a Python Data Analysis Library (NumFOCUS, Austin TX). Continuous data were summarized with means and standard deviations, and categorical data were summarized using percentages and counts for all athletes.

Participants who did not respond to any questions after the eligibility and demographics questions were excluded from the analysis.

### Content Analysis

Two authors (S.R., H.O.) used NVivo 12 (Lumivero, Burlington MA) to perform conventional content analysis [20] on participants’ responses to the open-ended questions: “Please elaborate on your experiences considering contraception as an endurance athlete. How did you feel throughout this process?” and “Please elaborate on your overall experience as an athlete discussing contraceptive options with your physician.” Each author reviewed the qualitative responses and coded emotion-based themes and topic-based subthemes (e.g., theme “frustrated” and subtheme “trial and error process”). Initially, approximately 25% of responses were analyzed independently. The authors then compared codes and codeveloped a set of themes and subthemes for the remainder of the thematic analysis. The rest of the responses were then coded independently, and subsequently the entire thematic analysis was reviewed together to ensure coding alignment. Frequency of central emotion themes and important quotations were presented.

## Results

### Athlete Demographics and Contraception Usage

A total of 473 individuals attempted to participate in the study, and 323 (68%) met the eligibility criteria. The average age was 26.6 years old [standard deviation (SD) 6.7], and the majority identified distance running as their primary sport (64%,* n* = 204) (Table 1). In terms of highest competition level reached, 20% (*n* = 65) represented their country at the international level, 45% (*n* = 142) competed at the national level, and 25% (*n* = 80) competed at the provincial or state level. Sixty-six percent (*n* = 213) of participants reached menarche between ages 12 and 14. Twenty-five percent (*n* = 80) did not experience menarche until 15 years of age or older, which would denote primary amenorrhea.

**Table 1:** Athlete demographics (*n* = 323)

**Mean age (years)**	26.6 (SD 6.7)
**Primary sport**	***n*** **(%)**
Distance running	204 (64)
Triathlon	32 (10)
Rowing	24 (8)
Distance cycling	21 (7)
Cross-country skiing	18 (6)
Distance swimming	9 (3)
Other	13 (4)
**Highest level of competition attained**	
International level	65 (20)
National level	142 (45)
Provincial level	80 (25)
Other	32 (10)

Hormonal IUDs (51%, *n* = 92) were the most currently used contraceptive by participants, while OCPs (87%, *n* = 157) were the most previously trialed method (Table 2). Thirty participants (11%) considered but did not start contraception requiring a prescription. Of those who currently used OCPs, 75% (*n* = 39) used combined pills, 11% (*n* = 6) used progestin-only pills, and 14% (*n* = 7) did not specify. Of those who previously used OCPs and switched to a different method (*n* = 96), 67% now use hormonal IUDs, 19% nonhormonal IUDs, 4% implants, 1% patches, 2% rings, and 3% permanent methods. Thirty-seven percent (*n* = 67) of athletes who currently used a contraceptive have only tried one method.

**Table 2:** Contraception usage history (*n* = 286)

Type	Currently use, *n* = 182 (%)	Previously used, *n* = 180 (%)
Oral contraceptive pills (OCPs)	52 (29)	157 (87)
Hormonal intrauterine devices (IUDs)	92 (51)	22 (12)
Nonhormonal IUDs	24 (13)	19 (11)
Implants	7 (4)	5 (3)
Patches	1 (1)	6 (3)
Rings	2 (1)	19 (11)
Injections	0 (0)	3 (2)
Permanent methods	4 (2)	0 (0)
Considered but never started contraception	30 (11)	
Average number of contraceptives trialed (including different brands within the same category)	2.3 (SD 2)	

Not shown in Table 2, contraceptive usage was analyzed by age and highest level of sport attained. Current OCP users were on average 24.7 years old (SD 5.7), while current hormonal IUD users were 26.4 (SD 5.5) and current nonhormonal IUD users were 27.7 (SD 8.7).

Overall, 88% (*n* = 53) of international level, 88% (*n* = 108) of national level, and 91% (*n* = 68) of provincial level athletes had previously used or currently used contraceptives. International level athletes had lower rates of current use at 52% (*n* = 31), versus 67% for both national (*n* = 83) and provincial level (*n* = 50) athletes. Of those currently using contraceptives, hormonal IUDs were the most common across all levels of sport (45% international level, 51% national level, and 56% provincial level), followed by OCPs (32% international level, 28% national level, and 28% provincial level) and then nonhormonal IUDs (12% international level, 14% national level, and 14% provincial level).

Additionally, given that it may take 3–6 months for side effects to subside, contraceptives were analyzed by duration of use. For currently used methods, the average length of use was 4.6 years (SD 5.0) for OCPs, 2.8 years (SD 2.5) for hormonal IUDs, and 2.9 years (SD 3.3) for nonhormonal IUDs. For previously used contraceptive options, the average length of use before stopping was 3.7 years (SD 5.0) for OCPs, 4.3 years (SD 3.0) for hormonal IUDs, and 1.7 years (SD 1.3) for nonhormonal IUDs. The analysis of the distribution of participants’ time spent on previous contraceptive methods revealed modes of 4.6 years for hormonal IUDs, 0.7 years for nonhormonal IUDs, and 1.0 year for OCPs.

### Experiences on Current Contraceptives

For the following section, the analysis focused on the three most commonly used contraceptives among participants: OCPs, hormonal IUDs, and nonhormonal IUDs.

#### Positive and Negative Side Effects

**Fig. 1:**
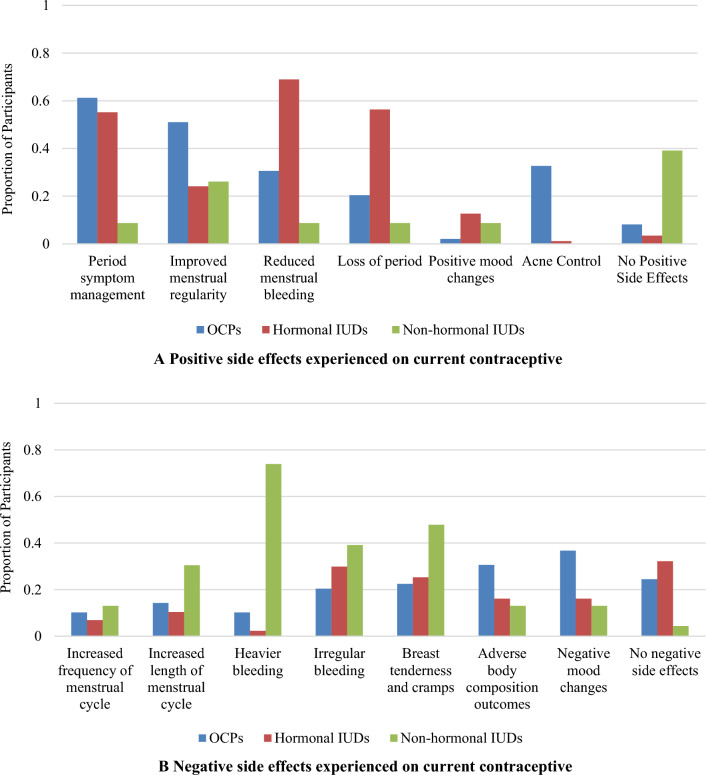
**A** Positive side effects experienced on current contraceptive (OCPs* n* = 49, hormonal IUDs *n* = 87, nonhormonal IUDs* n* = 23). **B** Negative side effects experienced on current contraceptive (OCPs *n* = 49, hormonal IUDs* n* = 87, nonhormonal IUDs *n* = 23)

Sixty-nine percent (*n* = 60) and 56% (*n* = 49) of hormonal IUD users experienced reduced or complete loss of menstrual bleeding, respectively (Fig. 1A). Fifty-one percent (*n* = 25) of OCP users benefited from improved menstrual regularity, and 33% (*n* = 16) reported better acne control. Both hormonal IUDs (55%, *n* = 48) and OCPs (61%, *n* = 30) were associated with period symptom management. In comparison, nonhormonal IUD users did not experience as many positive side effects, with 39% (*n* = 9) reporting no positive effects. Similar trends for positive side effects were seen when participants were asked about contraceptives they previously used.

Regarding negative side effects participants experienced on their current method, 74% (*n* = 17) of nonhormonal IUD users reported heavier menstrual bleeding, and 48% (*n* = 11) breast tenderness (Fig. 1B). For OCP users, negative mood changes (37%, *n* = 18) and adverse body composition outcomes (e.g., weight gain, bloating) (31%, *n* = 15) were the most frequently experienced negative side effects. For hormonal IUD users, 30% (*n* = 26) experienced irregular bleeding and 32% (*n* = 28) did not experience any negative side effects.

#### Perceived Impact on Athletic Performance in Training and Competition

**Fig. 2:**
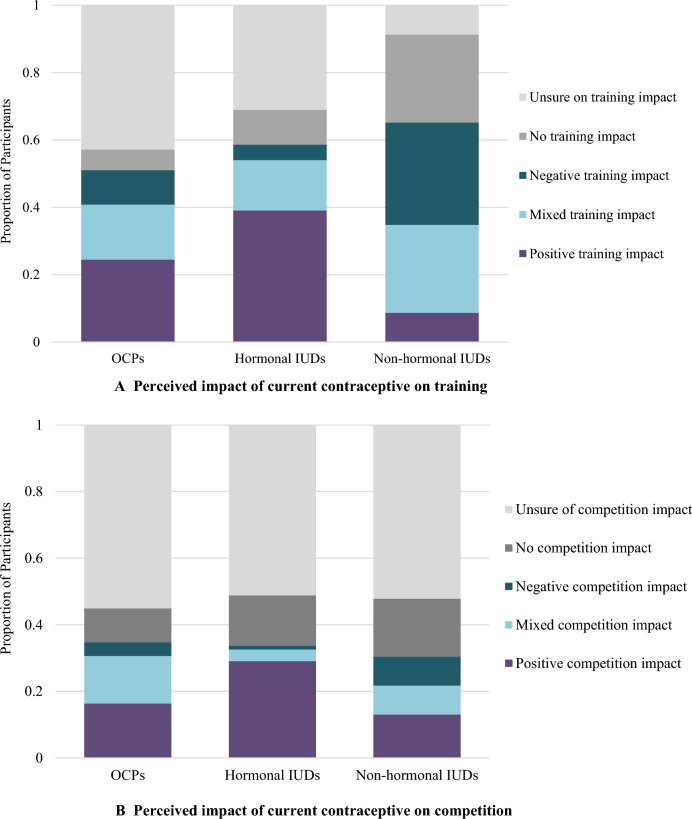
**A** Perceived impact of current contraceptive on training (OCPs *n* = 49, hormonal IUDs *n* = 87, nonhormonal IUDs *n* = 23). **B**. Perceived impact of current contraceptive on competition (OCPs *n* = 49, hormonal IUDs *n* = 86, nonhormonal IUDs *n* = 23)

Hormonal IUDs had the highest percentage of users who perceived positive training and competition impacts at 39% (*n* = 34) and 29% (*n* = 35), respectively (Fig. 2A, B). A minimal percentage of hormonal IUD users experienced a negative impact on training (4%, *n* = 4) or competition (1%, *n* = 1). In contrast, only 9% (*n* = 2) of nonhormonal IUD users reported a positive training impact and 30% (*n* = 7) reported a negative impact. OCP users perceived a more favorable training impact than those using the nonhormonal IUD: 24% (*n* = 12) experienced a positive impact, while 10% (*n* = 5) experienced a negative impact. More than half of all participants across each contraceptive category were unsure about the impact of their current contraceptive method on competition.

#### Happiness with Contraceptives

**Fig. 3:**
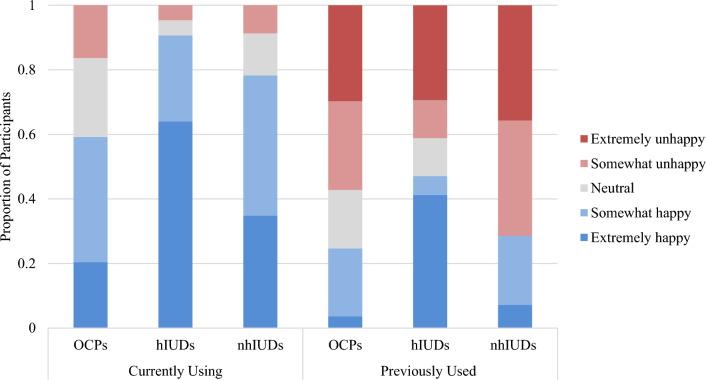
Degree of happiness with current and previously used contraceptives (currently using: OCPs *n* = 49, hormonal IUDs* n* = 86, nonhormonal IUDs *n* = 23; previously used: OCPs* n* = 138, hormonal IUDs *n* = 16, nonhormonal IUDs *n* = 14)

Participants endorsed higher levels of happiness with their current method compared with previous method(s) across all forms of contraceptives (Fig. 3). Current users of hormonal IUDs had the highest level of “extremely” (64%, *n* = 55) and “somewhat happy” (27%, *n* = 23) responses, and only 5% (*n* = 4) “neutral” and 5% (*n* = 4) “somewhat unhappy” responses. For current OCPs users, 20% (*n* = 10) were “extremely happy,” 39% (*n* = 19) were “somewhat happy,” 24% (*n* = 12) were “neutral,” and 16% were “somewhat unhappy” with their contraceptive. For nonhormonal IUD users, 35% (*n* = 8) were “extremely happy,” 43% (*n* = 10) were “somewhat happy,” 13% (*n* = 3) were “neutral,” and 9% (*n* = 2) were “somewhat unhappy.” These results align with responses on whether participants would recommend their contraceptive to a fellow female endurance athlete, as hormonal IUDs had the highest rate of recommendation (83% for currently using, 41% for previously used).

Given that OCPs were the most common previously used method, and hormonal IUDs were the most common currently used method, the happiness of participants (*n* = 61) who switched from OCPs to hormonal IUDs was examined. Happiness closely mirrored data trends seen in Fig. 3, with 67% of participants “extremely happy” with their current IUD while only 2% were extremely happy with their previous OCP.

### Contraceptive Consideration Process

Pregnancy prevention was the most common reason participants initially considered contraception (85%, *n* = 245), followed by regulation of menstrual cycle timing (41%, *n* = 119) and menstrual symptoms (41%, *n* = 118), and reducing heavy bleeding (26%, *n* = 75) (Table 3A).

**Table 3:** Summary of contraception consideration process (*n* = 289)

A. Reasons for considering contraception *n* (%)	
For pregnancy prevention	246 (85)
To regulate menstrual cycle (in relation to athletic performance)	119 (41)
To reduce menstruation symptoms	118 (41)
To reduce heavy bleeding	75 (26)
For acne control	61 (21)
To induce first period	7 (2)
Other	13 (5)

B. Positive side effects considered	
Period symptom management	153 (53)
Reduced menstrual bleeding	148 (51)
Improved cycle regularity	144 (50)
Loss of period	101 (35)
Acne control	60 (21)
Positive mood changes	30 (10)
I did not consider positive side effects	33 (11)

C. Negative side effects considered	
Adverse body composition outcomes	202 (70)
Negative mood changes	151 (52)
Increased fatigue	109 (38)
Heavier bleeding	79 (27)
Irregular bleeding	78 (27)
Breast tenderness and cramps	76 (26)
Increased frequency of menstrual cycle	56 (19)
Increased headaches	56 (19)
Increased length of menstrual cycle	50 (17)
Nausea/vomiting	48 (17)
I did not consider negative side effects	25 (9)

In the contraceptive consideration process, over half of participants factored positive side effects such as period symptom management (53%, *n* = 153), improved menstrual regularity (50%, *n* = 144), and reduced menstrual bleeding (51%, *n* = 148) into their decision (Table 3B). The most common negative side effects considered included adverse body composition changes (70%, *n* = 202), negative mood changes (52%, *n* = 151), and increased fatigue (38%, *n* = 109) (Table 3C). Other related factors included ease of use (81%, *n* = 235), efficacy of pregnancy prevention (78%, *n* = 226), and cost (35%, *n* = 102).

Of participants who considered but ultimately decided not to use a prescribed contraceptive, 63% (*n* = 19) stated this was because they were worried it could impact their athletic performance.

**Fig. 4:**
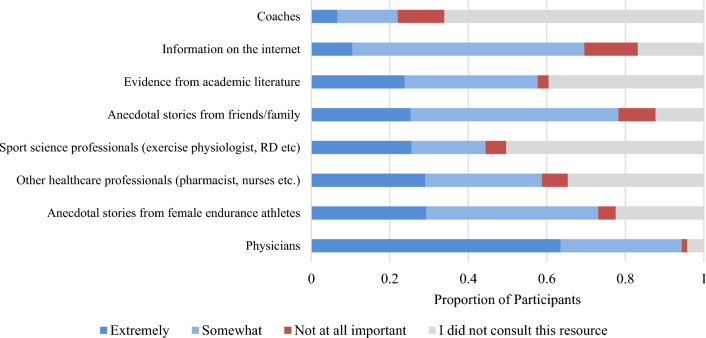
Importance of different information sources in contraception consideration process (*n* = 285)

Female endurance athletes valued different information sources in the selection process (Fig. 4). Physicians were ranked the most important resource, with 64% (*n* = 181) indicating their input was “extremely important.” The least consulted resources included coaches, sports scientists, and evidence from academic literature, with 66% (*n* = 189), 50% (*n* = 144), and 40% (*n* = 113) of participants never having consulted these resources, respectively.

Over half of participants “somewhat” (34%, *n* = 98) or “strongly” (18%, *n* = 51) felt they did not have enough information to make an informed decision about which contraceptive would be best for them as an athlete.

#### Open-Ended Responses

**Fig. 5:**
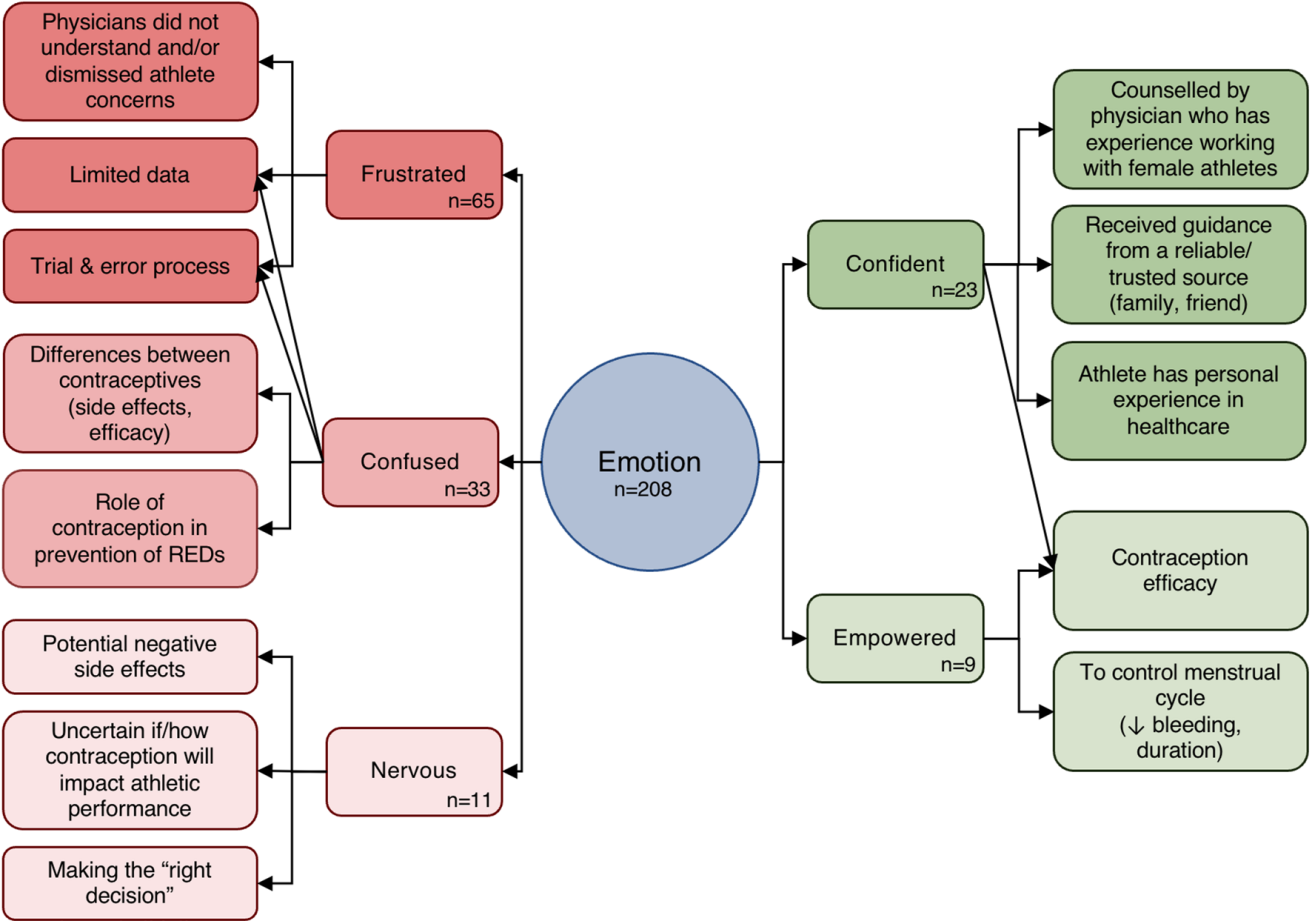
Subthemes surrounding the five most common emotions athletes experienced in the contraception selection process

Thirteen subthemes were identified in the open-ended responses about emotions athletes experienced in the contraception selection process (Fig. 5; Table 4). Frustration (*n* = 65/208) was the most common emotion athletes experienced when selecting a contraceptive, mainly because limited data reinforces a trial-and-error approach. Some participants also expressed frustration with the counseling they received from physicians as they felt their physicians did not understand and/or dismissed their concerns. Confusion (*n* = 33) was the second most frequently reported emotion, relating to differences between contraceptives in terms of side-effect profiles, efficacy, and impact on sport. Athletes also felt confused about the role contraception plays in the prevention and/or masking of relative energy deficiency in sport (REDs). Several athletes reported feeling nervous (*n* = 11) about potential negative side effects and noted it is challenging to make the “right” contraceptive decision when it is not possible to predict if or how athletic performance will be impacted.

**Table 4:** Summaries of open-ended responses about emotions experienced in the contraception selection process (*n* = 208)

Emotion themes	Subthemes	Sample quotes
Frustrated (*n* = 65)	Limited data	“Finding a contraceptive option as a high-level endurance athlete is extremely frustrating. There is not enough information out there as to the potential effects hormonally on the body from different contraception. As an athlete who has always had a period, I felt limited in my options because I didn’t need contraception to regulate my period.”
	Trial and error	“Most of the information was anecdotal, and what works well for one person may not be the case for others. The trial-and-error method to figure out what works for me took 6 months to a year when I started—if there is a way to improve information access to reduce that ‘guessing game’ window that would have been fantastic 10 + years ago.”
	Physician did not understand and/or dismissed athlete concerns	“It’s frustrating. They don’t understand how small impacts are amplified in endurance sports. I felt like I was dismissed and told it doesn’t make a difference since it doesn’t for the average patient.”
Confused (*n* = 33)	About differences between contraceptives	“Overall, I felt confused by all the different options and trying to figure out which would be best for me. I didn't find the healthcare professionals in my life very helpful when considering my sport in relation to contraceptives.”
	About role of contraception in prevention of REDs	“Physician said I should take birth control to regulate my period (was having periods as infrequently as every 5–6 months) which I did not question at the time, but now I know that this was most likely a symptom of REDs and taking birth control to menstruate would have been masking the issue instead of addressing the real cause.”
Confident (*n* = 23)	Counseled by physician who has experiencing working with female athletes	“I was lucky to be able to consult my physician, who works with female athletes to make the best contraceptive choice for myself. I know that not everyone has that luxury and I think it made me feel much more at ease and confident in my decision to take contraceptives as an endurance athlete.”
	Received guidance from a reliable/trusted source	“I had the most guidance from my mother, who shared her experiences with the pill and the hormonal IUD with me. That gave me a lot of confidence.”
	Athlete has personal experience in healthcare	“I’m a physician so I felt comfortable with my choice – specifically wanted a hormonal IUD to reduce severity of menstrual bleeding/cramping and for localized rather than systemic hormones. Ease of use and effectiveness in pregnancy prevention were high priorities for me because I knew the data but impacts on performance would have been meaningful if data were available!”
Nervous (*n* = 11)	Potential negative side effects	“Anxious about potential side effects (i.e. mood, weight), especially when I thought that those giving me the information about different contraceptive options did not take into account that maintaining performance in sport was a priority for me.”
	Uncertain how contraceptive will impact athletic performance	“I honestly felt somewhat afraid of using contraception on the chance that it could negatively affect my athletic performance. I had heard several horror stories from my fellow athletes regarding contraception and how it had resulted in extra weight gain, mood swings, acne, etc. This was not one single type of contraception either. I had to thoroughly weigh out my options and what I felt was most important to me at the time. Being unprotected with sex or exceeding my goals athletically.”
	About making the “right” decision	“It was unclear on how my body composition would change—which ultimately impacts my sport (W/kg) and how I view myself. If I don’t feel good, it negatively impacts the frequency of training as well as intensity. I could blame not feeling great on something other than myself, which would give me too many outs or reasons for not trying my best or pushing myself into the uncomfortable zone.”
Empowered (*n* = 9)	About contraceptive efficacy	“I was excited for peace of mind and the effectiveness of contraception.”
	To control menstrual cycle (decrease heavy bleeding, duration)	“My initial reason for taking the pill was solely because over-the-counter painkillers were not effective for managing my cramps. The pill has helped me in that regard, and I appreciate the convenience of never being caught off guard by my period because of how much it can throw off a week of training.”

Not all athletes felt negative emotions during the selection process. Those who felt confident (*n* = 23) said they were counseled by a physician who had experience working with female athletes, received guidance from a reliable/trusted source (friend, family member), and/or had personal experience working in healthcare. Feelings of empowerment stemmed from knowledge about contraception efficacy, and the ability to regulate their menstrual cycle and premenstrual symptoms with contraception.

### Physician Counseling

**Fig. 6:**
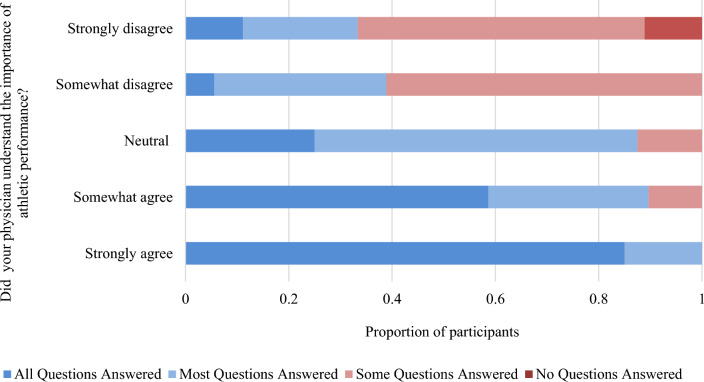
Proportion of questions that participants felt were answered by their physician stratified by how strongly the participant felt their physician understood the importance of athletic performance in their discussion (strongly agree *n* = 20, somewhat agree *n* = 29, neutral *n* = 8, somewhat disagree *n* = 18, strongly disagree *n* = 9)

Eighty-six percent (*n* = 211) of participants discussed contraception with a physician. Of these, 56% (*n* = 114) consulted their family physician, 31% (*n* = 63) an obstetrician/gynaecologist, and 5% (*n* = 11) a sports medicine physician. Less than half of participants (41%, *n* = 84) discussed athletic performance in relation to contraception with their physician (Table 5B). Of those that did, 24% (*n* = 20) “strongly agreed” and 35% (*n* = 29) “somewhat agreed” that their physician factored the participant’s value of athletics into the contraceptive discussion (Table 5C). One-third were not satisfied with their physician’s contraceptive counseling ability in the context of athletic performance (21%, *n* = 18 for somewhat, 11%, *n* = 9 for strongly), and 34% (*n* = 68) sought out additional information as a result (Table 5D). The more strongly participants felt like their physician understood the value they place on athletic performance, the more likely they were to feel like all their questions were answered (Fig. 6).

**Table 5:** Contraception counseling experiences with physicians

A. Was the physician able to answer your questions about contraceptive options? (*n* = 205)	*n* (%)
All my questions were answered	93 (45)
Most of my questions were answered	65 (32)
Some of my questions were answered	44 (22)
None of my questions were answered	3 (2)

B. Did you talk about athletic performance in relation to contraception in your discussion with your physician? (*n* = 205)	*n* (%)
Yes	84 (41)
No	121 (59)

C. Did you feel like your physician factored the value you place on athletic performance into your discussion? (*n* = 84)	*n* (%)
Strongly agree	20 (24)
Somewhat agree	29 (35)
Neutral	8 (10)
Somewhat disagree	18 (21)
Strongly disagree	9 (11)

D. Did you ever seek out additional information from other sources because you were not satisfied with your physician's contraceptive counseling ability? (*n* = 203)	*n* (%)
Yes	68 (34)
No	78 (38)
I did seek out other sources, but not because I wasn’t satisfied with my physician	57 (28)

## Discussion

This exploratory study is the first to quantify and qualify the lived experiences of female endurance athletes with usage and selection of the three most common forms of contraception: OCPs, hormonal IUDs, and nonhormonal IUDs. Novel insights were gained regarding the perceived impact of different contraceptives on performance in training and competitions, factors athletes considered in the selection process, and the important role physicians play in contraceptive counseling.

### Contraceptive Usage

Hormonal IUDs (51%) were the most common contraceptives currently used by female endurance athletes in this study. This was followed by OCPs (29%) and nonhormonal IUDs (13%), while 10% implemented no contraceptive. This finding is in contrast with studies focused on the general female athlete population, which demonstrate higher rates of OCPs compared with hormonal IUDs [7, 8, 12, 13]. Baumgartner et al. (2023) did find that athletes who participated in “lean sports” (which included endurance sports) were more likely to use hormonal IUDs than in “non-lean sports” [17]. Historically, OCPs have been the most frequently used method among athletes and the general population [21, 22]. The contraceptive usage data from this study suggest a shift away from OCPs toward hormonal IUDs among endurance athletes. Two-thirds of participants that previously used OCPs and switched to another method chose hormonal IUDs. This may be explained by broader trends in prescribing practices among healthcare professionals, as IUDs are now a first-line contraceptive option for nulliparous women [23]. There may also be a conscious effort to move toward local hormone release given common perceived, but not scientifically validated, concerns of systemic hormonal effects such as adverse body composition changes in the endurance athlete population.

It should be noted that, while 10% of athletes in this study had never used contraceptives, this does not represent the rate of nonusage in the endurance community as the eligibility criteria required athletes to have considered using contraception to participate.

#### Hormonal IUDs

Hormonal IUDs were the most common and best-tolerated contraceptive method for female endurance athletes in this study. This is a key finding as there are no previous experimental studies on the impact of hormonal IUDs on athletic performance [11]. Over 90% of current hormonal IUD users were “extremely” or “somewhat happy.” Less than 5% of current users experienced a negative impact on training, while nearly 40% thought their hormonal IUD had a positive training impact. A similarly favorable pattern was seen for perceived competition impact. Two-thirds of individuals using hormonal IUDs had previously used OCPs. For these participants, happiness (rated as “somewhat” or “extremely happy”) with their contraceptive increased from 30% on OCPs to 90% with hormonal IUDs. These results are not surprising given the low frequency of concerning negative side effects participants experienced such as adverse body composition outcomes and negative mood changes, and high rates of positive side effects such as decreased menstrual bleeding and period symptom management. Minor negative side effects reported included irregular bleeding possibly due to inconsistent menstrual suppression and breast tenderness. While there are several studies that have examined the perceived side effects of hormonal contraceptives in athletes, most did not specifically examine the impacts of IUDs. Martin et al. (2018) observed a high rate (83%) of reported positive symptoms among athletes using hormonal IUDs; however, their study primarily focused on OCP users and had a small number of hormonal IUD users (*n* = 6) [13].

These findings are particularly significant as there is a high prevalence of female athletes with heavy menstrual bleeding, which is associated with anemia, an increased use of iron supplementation, and reported negative impacts on performance [24, 25]. In the literature, hormonal IUDs have been shown to improve both heavy menstrual bleeding and dysmenorrhea to varying extents [1, 26, 27]. Over time, some women may lose their period completely on a hormonal IUD [28]. Amenorrhea or even decreased blood loss can help those with iron deficiency anemia recover their hemoglobin levels, and in turn, improve their performance [29, 30]. One disadvantage of these menstrual cycle changes is that it may be more challenging for athletes to recognize if they are fueling adequately for their level of training or developing REDs [31]. This topic is further explored in Sect. 4.4.

#### OCPs

In this study, OCPs were the second most commonly used and well-tolerated method after hormonal IUDs. Greater than 75% of OCP users were on a combined estrogen–progestin type. Participants’ perceived impact of OCPs on training varied: over 20% of athletes felt their OCP had a positive impact, 10% a negative impact, 15% mixed, 5% no training impact, and over 40% of athletes were unsure. Impact on competition showed a similar distribution, although there was even more uncertainty (55%) about effects. This wide distribution may be explained by the positive and negative side effects athletes experienced, the magnitude of these side effects, and how these trickle down to sport.

The most common positive side effects participants experienced on OCPs were period symptom management (reduced cramping) and improved menstrual cycle regularity (decreased bleeding, shorter more predictable cycle). Both have been extensively documented in athletes and the general population and can make a significant difference for women with heavy menstrual bleeding, dysmenorrhea, endometriosis, and polycystic ovarian syndrome [13, 32]. Another possible benefit of OCP use among athletes is the ability to manipulate the menstrual cycle via skipping the pill-free week [13, 33]. Although this benefit was not qualitatively investigated in this study, Oxfeldt et al. (2020) demonstrated that 60% of elite athletes who use OCPs will manipulate their cycle [7]. This may be beneficial for athletes who want to avoid menstrual bleeding or symptoms during important competition or training periods.

Conversely, over 30% of individuals on OCPs experienced adverse body composition outcomes (weight gain, bloating) and negative mood changes (irritability, depression), which were the two most concerning negative side effects that participants considered during the selection process. Ekenros et al. (2022) reported comparable rates of mood-related side effects (41%) among athletes who used OCPs, though reported lower rates (20%) of weight gain [14]. This difference in perceived body composition changes may stem from study population differences, as endurance athletes are more likely to be weight-focused given the importance of weight for performance, compared with athletes from a broad range of sports (including weightlifting, equestrian, handball, etc.).

Clinical studies investigating OCPs and weight gain are contradictory [34–36], and both weight gain and weight loss are listed as possible side effects of hormonal contraceptives. In endurance sport, even a perceived increase in weight and/or undesired body composition changes could have a negative impact on performance. With respect to mood, some studies have demonstrated an association between combined OCP use and negative mood changes, although it depends on the type [37]. Negative mood changes could certainly negatively impact training via amotivation or anhedonia, and competition via poor mental performance on race day.

#### Nonhormonal IUDs

Nonhormonal IUDs do not appear to be as well tolerated as hormonal IUDs by participants in this study. Over 70% of nonhormonal IUD users experienced heavier and more irregular bleeding. This finding is consistent with studies in the general population [38]. For example, one study that explored the side effects of nonhormonal IUDs over time reported that 67% (*n* = 6440) of users experienced increased menstrual blood loss in the first 9 weeks following insertion. This percentage decreased to 48% at > 39 weeks post insertion [39]. In this study, when asked to describe how the side effects changed over time, some athletes said the heavier bleeding and cramping gradually subsided but were still worse than before insertion, while others felt there was no change. Participants consistently cited increased bleeding as having a major negative impact on training and competition, and it led to early removal in some cases. For participants who discontinued using a nonhormonal IUD, the most common duration was approximately 1 year. In contrast, hormonal IUD users typically used them for about 5 years, aligning with the standard prescribed duration. This suggests that nonhormonal IUD users were more likely to remove their IUDs early, likely due to side effects, compared with those using hormonal IUDs.

Despite the above, many participants expressed a desire for a nonhormonal contraceptive. Reasons for this are multifactorial—some mentioned fear of potential side effects relating to exogenous hormones, specifically body composition changes, though there is a lack of evidence for this in the literature [40]. Other participants expressed a desire to continue to menstruate as a regular cycle can function as a monthly marker of adequate energy availability. Over 75% of female endurance athletes currently using nonhormonal IUDs were “extremely” or “somewhat happy,” suggesting that it is a good contraceptive option for those who tolerate it. For example, athletes who have a lighter cycle with minimal cramps may respond more favorably than athletes with a history of heavy menstrual bleeding and/or dysmenorrhea. Athletes should be counseled about the possible side effects of heavier bleeding and cramping to make an informed decision.

#### Other Contraceptive Methods

There were a low number of participants reporting use or consideration of implants, patches, rings, injections, and permanent methods; therefore, it is challenging to draw practical conclusions about their perceived performance impacts from these data. It should be noted that no participants in this study currently used the depot-medroxyprogesterone acetate injection. This is not surprising as this is the only contraceptive that has evidence of weight gain in the literature [41]. Additionally, the injection is associated with bone mineral density loss with prolonged use, which is reversible to a certain extent upon discontinuation [42]. Physicians are aware of these potential side effects and may be less likely to recommend and/or prescribe the injection to female endurance athletes as a result. If the injection is presented as an option, athletes may be less likely to select it when counseled about the risks as both weight gain and bone mineral loss may have a negative impact on performance.

### Contraception Selection Process

The main factors participants considered in their decision included contraception efficacy and ease of use, menstrual cycle control, and avoidance of potential negative side effects such as body composition changes, negative mood changes, and increased fatigue. These side effects may be detrimental to optimal athletic performance. Martin et al. (2018) also identified weight gain and mood changes as top concerns among the general elite athlete population before initiating contraception [13].

Participants consulted a range of resources in the selection process. Input and insights from physicians were most highly valued. Athletes also valued input from other healthcare professionals including nurse practitioners, pharmacists, sport scientists, and registered dietitians, although fewer athletes had access to their expertise. Over 70% of participants also relied on anecdotal stories from teammates, friends, and family members. This is expected given there are limited available data on which contraceptives endurance athletes are using and their impact on performance. Coaches were the least consulted resource with only 34% of athletes talking to their coach and only 7% feeling that it was “extremely important” in their contraceptive selection process. Oxfeldt et al. (2020) similarly found that only 13% of athletes had spoken to their coaches about their contraceptive choice [7]. This may be because athletes perceived their coaches did not have the knowledge to counsel appropriately or because they were not approachable for discussions of this nature. Female coaches also continue to be underrepresented in the endurance community [43, 44].

Overarching themes identified in the qualitative section of this study showed that decision-making is often the result of trial and error. Athletes expressed frustration that there is limited scientific evidence to guide their decision and felt they could not predict how their body would respond. Participants tried an average of 2.3 different contraceptives (including different methods and brands). This trial-and-error process is not unique to athletes as the 2020 Kaiser Family Foundation’s “Women’s Health Survey” reported a lifetime average of 3.4 methods/brands. This may be due to a lack of research in the field of reproductive health, poor contraceptive counseling, and/or personal factors such as changing priorities and health.

### Physician Counseling

Physicians have an important role to play in contraception counseling. Ninety-five percent of female endurance athletes in this study reported that information and insight from physicians was “extremely” or “somewhat” important to their decision. Despite this, 59% of athletes who received contraception counseling by a physician did not talk about athletic performance in relation to contraception. Among the participants who did receive counseling in the context of sport, 36% did not feel like their physician adequately considered athletic performance in the discussion. This highlights that a high proportion of endurance athletes do not feel understood by their physician. This sentiment is succinctly captured in the following open-ended response: “*It’s frustrating. They don’t understand how small impacts are amplified in endurance sports. I felt like I was dismissed and told it didn’t make a difference since it doesn’t for the average patient*.”

Physicians’ openness to discussion and comfort level with both contraception and sport are factors that related to the perceived quality of counseling provided. Participants who were counseled by female physicians with personal experiences in sport cited increased confidence with their contraceptive decisions. Athletes who felt athletics were adequately factored into the discussion were more likely to feel like all of their questions were answered. Patient-centered care has been linked to increased patient satisfaction and perceived quality of care in healthcare, and thus an individualized approach to counseling is crucial [45]. For example, counseling may differ for a young athlete seeking oral contraceptive pills for acne control, versus relief from painful premenstrual symptoms, or contraception only.

### REDs and Contraception

The International Olympic Committee defines REDs as: “a syndrome of impaired physiological and/or psychological functioning experienced by female and male athletes that is caused by exposure to problematic (prolonged and/or severe) low energy availability. The detrimental outcomes include, but are not limited to, decreases in energy metabolism, reproductive function, musculoskeletal health, immunity, glycogen synthesis and cardiovascular and hematological health, which can all individually and synergistically lead to impaired well-being, increased injury risk and decreased sports performance” [46]. While characterization of REDs concerns in relation to contraception was not an initial objective of the survey, the qualitative open-ended response data indicated that this is a significant topic of concern for female endurance athletes.

Specifically, participants were concerned that starting a hormonal contraceptive may impact their ability to monitor for signs of REDs using their menstrual cycle. Amenorrhea is one of the most obvious indicators of REDs and a common side effect of hormonal contraception [46]. For athletes who become amenorrheic while using hormonal IUDs or OCPs there are other symptoms besides menstruation they can use to monitor their risk. These include but are not limited to fatigue, rapid weight loss, frequent illness, irritability and depression, performance and training plateaus or declines, and bone stress injury [46, 47]. Athletes should see a physician for REDs screening and management if they are concerned. Changes in cholesterol and resting metabolic rate can assist with the diagnosis, as well as a significant change in sex hormones (estrogen, LH, FSH, testosterone) for those not on hormonal contraception.

For athletes with REDs who are not on hormonal contraception, it is not recommended to prescribe OCPs to “treat” primary or secondary amenorrhea [48]. OCP withdrawal bleeds are not equivalent to a natural menstrual cycle; therefore, this intervention does not correct the underlying etiological cause of REDs. If the energy deficit is not corrected, the attainment of peak bone density in young athletes may be compromised [48]. In oligomenorrheic athletes, there is some evidence that the 17β-oestradiol transdermal patch given continuously with cyclic oral micronized progesterone increases bone mineral density scores compared with those randomized to combined OCPs or no treatment [49]. Overall, further research exploring REDs in relation to hormonal contraception is needed.

## Strengths and Limitations

Strengths of this study are that it comprehensively explores the female endurance athlete experience with contraception and includes the athlete voice. The relatively large sample size for a mixed-methods study, with 65% having competed at the national level or higher in their sport and 21% at the international level, is also a strength.

A main limitation is that the survey was exploratory in nature and no conclusions can be made regarding one contraceptive over another. Additionally, the study assessed the perceived overall impact of different contraceptive methods on athletic performance, rather than objective measurements. As there are many factors that may contribute to performance in training and competition (sleep, nutrition, stress, mental health, injuries, training plan, environmental factors, etc.), it can be difficult for participants to isolate the effects of contraception alone. The retrospective study design created the possibility of recall bias, especially for athletes who have tried multiple contraceptives or have used their current method for a long time. Length of time on one contraceptive may have also impacted how participants perceived its impact on sport as side effects can change or subside over time.

Restricting eligible participants to age 18 and older limits the generalizability of the findings to younger athletes. Participants were asked about their history of contraceptive use; thus, the data included experiences from those who started contraception before age 18, but this information was not stratified. There was no restriction of participation by nationality, and the study did not inquire about country of contraceptive prescription in the demographics section; therefore, it was not possible to evaluate whether cultural differences and/or contraceptive availability and cost influenced selection. Finally, as with many online survey studies, guaranteeing the authenticity of all participants was not possible; however, this risk was mitigated by clearly outlining the eligibility criteria on all recruitment materials and at the start of the survey.

## Conclusions

This study is the first to characterize the lived experiences of female endurance athletes with different contraceptive options. Hormonal IUDs were the most commonly used contraceptive method, appeared to be the best tolerated, and had the most positive perceived athletic impacts. This is a key finding as there is no other literature to date on the impacts of hormonal IUDs on endurance performance. In contrast, OCPs and nonhormonal IUDs were less well tolerated, with higher perceived negative side effects and performance impacts. Frustration was the most common emotion athletes experienced when selecting a contraceptive, mainly due to the trial-and-error process, lack of evidence, and frequent feelings of dismissal from physicians regarding athletic concerns. Conversely, athletes whose physicians provided counseling in the context of sport were more likely to feel confident and well informed in their decision. Physicians and other healthcare professionals play a vital role in addressing these issues and meeting the needs of female endurance athletes in the realm of contraception. A patient-centered approach, coupled with being well informed, is key to effectively counseling and supporting athletes in making informed decisions about their reproductive health.

## Footnotes

1 While there are other forms of contraceptives that can be obtained over the counter (e.g., condoms, spermicide etc.), use of the term “contraceptives” in this paper will refer to prescribed contraceptives methods.

2 Progestin and estrogen are the active hormonal components in combined OCPs, transdermal patches, and vaginal rings, whereas the progestin-only OCPs, hormonal IUDs, injections, and implants contain exclusively progestin.

## Declarations

### Data Availability

Data are available upon reasonable request.

## Abbreviations

FSH: Follicle-stimulating hormone
IUDs: Intrauterine devices
hIUDs: Hormonal intrauterine devices
nhIUDs: Nonhormonal intrauterine devices
LH: Luteinizing hormone
NCAA: National Collegiate Athletics Association
OCPs: Oral contraceptive pills
REDs: Relative energy deficiency in sport
